# Supplementary material for: Edge effects on tree architecture exacerbate biomass loss of fragmented Amazonian forests
Source: Nat Commun. 2023 Dec 14;14:8129. doi: 10.1038/s41467-023-44004-5 (PMC10721830; doi:10.1038/s41467-023-44004-5)
Supplement: Supplementary file 1 — Supplementary Information [file 41467_2023_44004_MOESM1_ESM.pdf]

Edge effects on tree architecture exacerbate biomass loss of fragmented Amazonian forests

AUTHOR LIST

Matheus Henrique Nunes [1, 2, 16], Marcel Caritá Vaz [3], José Luís Campana Camargo [4,5], William F. Laurance [6], Ana de Andrade, [5] Alberto Vicentini [5, 7], Susan Laurance [6], Pasi Raumonen [8], Toby Jackson [9], Gabriela Zuquim [10], Jin Wu [11], Josep Peñuelas [12, 13], Jérôme Chave [14], Eduardo Eiji Maeda [1, 15, 16]

AFFILIATIONS

[1] Department of Geosciences and Geography, P.O. Box 68, FI-00014 University of Helsinki, Helsinki, Finland

[2] Department of Geographical Sciences, University of Maryland, College Park, MD 20742, United States of America

[3] Institute for Environmental Science and Sustainabilty, Wilkes University, 84 W. South St. Box 91, Wilkes-Barre, PA 18766, U.S.A.

[4] Ecology Graduate Program, National Institute for Amazonian Research, (INPA), Manaus, Brazil

[5] Biological Dynamics of Forest Fragments Project (BDFFP) at National Institute for Amazonian Research (INPA), Manaus, Brazil.

[6] Centre for Tropical Environmental and Sustainability Science, College of Science and Engineering, James Cook University, Cairns, Queensland 4878, Australia

[7] Coordenação de Pesquisas em Ecologia, Instituto Nacional de Pesquisas da Amazônia (INPA), Manaus, AM, Brasil

[8] Computing Sciences, Tampere University, Korkeakoulunkatu 3, 33720, Tampere, Finland

[9] Plant Sciences and Conservation Research Institute, University of Cambridge, Cambridge, CB2 3QZ, United Kingdom

[10] Amazon Research Team, Department of Biology, University of Turku, Finland

[11] School of Biological Sciences and Institute for Climate and Carbon Neutrality, The University of Hong Kong, Pokfulam Road, Hong Kong, China

[12] CREAF, Cerdanyola del Vallès, Barcelona 08193, Catalonia, Spain.

[13] CSIC, Global Ecology Unit CREAF-CSIC-UAB, Bellaterra, Barcelona 08193, Catalonia, Spain.

[14] Laboratoire Evolution et Diversité Biologique, CNRS, UPS, IRD, Université Paul Sabatier, Toulouse, France

[15] Finnish Meteorological Institute, FMI, Helsinki, Finland

[16] Corresponding authors:

Matheus Henrique Nunes: mhnunes@umd.edu

Eduardo Eiji Maeda: eduardo.maeda@helsinki.fi

SUPPLEMENTARY MATERIAL

Supplementary methods 1: Quantitative structure modelling

QSMs were generated with TreeQSM v.2.4.0 (https://github.com/InverseTampere/TreeQSM). The tree point clouds were initially filtered to remove leaves and noise using the filtering function in the TreeQSM package using MATLAB. Low point-density regions with points whose spherical neighbourhood of 3 cm radius contained less than 4 points were removed. Small separate regions of the point cloud were also removed by covering the points with patches of size 10-20 cm in diameter with overlapping neighbours up to 13 cm from the patch centres and removing the clusters of patches that had less than 3 overlapping neighbours. Finally, some leaves and small branches were removed based on the number of shortest paths. The point cloud was covered with patches whose minimum diameters were 2-4 cm and the maximum diameter was based on a rough DBH estimate and then the shortest paths from each patch to the tree base were defined. This yields information on how many shortest paths go through every patch - most paths are close to the tree base and only one or few paths near the branch tips. Thus, we removed all the patches whose

number of paths going through them was sufficiently low compared to the path length: below 15 at the maximum path lengths and linearly smaller threshold for smaller path lengths. We ensured that there was a path to every branch tip and that patches with sufficiently high path numbers had always their neighbourhoods included. The QSM generation with TreeQSM needed the optimization of the input parameters PatchDiam1, PatchDiam2Min, and PatchDiam2Max. For each tree a few values for these parameters were selected based on the rough DBH estimate. Then we generated QSMs with all different parameter combinations and finally selected the optimal input values by minimising the average point-model-distance (Supplementary Figure 1).

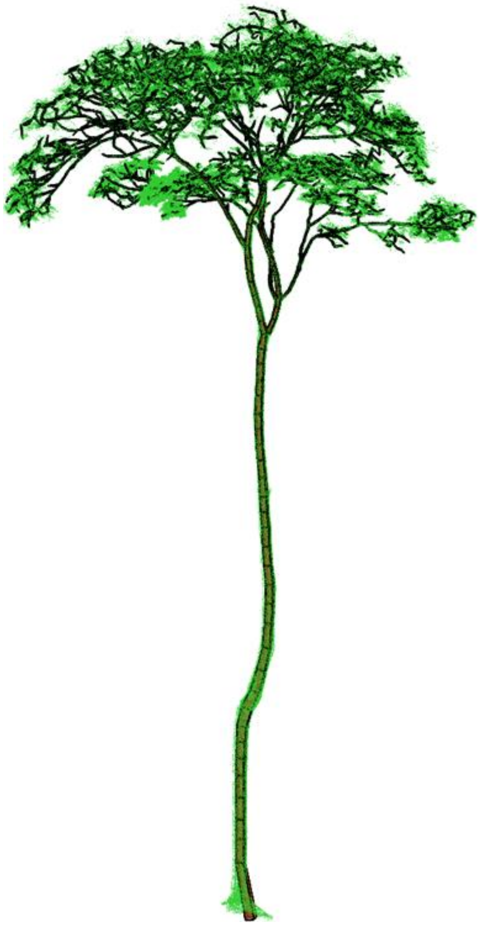

Supplementary Figure 1. Quantitative structure modelling (QSM) of a tree (in this example, belonging to the species *Eschweilera truncata*) in Central Amazonia. Green points represent the tree point cloud obtained using high-resolution terrestrial laser scanning (TLS). The fitted brown cylinders represent the tree QSM used to estimate woody volume and architectural traits.

Supplementary methods 2: Estimation and ecological significance of architectural traits

We calculated six architectural traits using established methods and explain their ecological significance. A distribution of values for each architectural trait can be found in Supplementary Figure 2.

Surface area per unit volume: Despite the importance of surface area per unit volume to understand vegetation dynamics, traditional methods to quantify woody surface area are highly laborious. It requires destructive sampling to cut up trees into components for direct measurements of surface areas[1]. TLS provides direct measurements of surface area and volume compartmentalised into stems and branches, without the need of destructive sampling. The reconstruction of surfaces and volume are described in detail in

<https://github.com/InverseTampere/TreeQSM/blob/master/Manual/TreeQSM_documentation.pdf>.

The amount of surface area per unit volume is primarily determined by the average diameter of branches and stems, despite surface irregularities that may affect this relationship. Although surface area and volume are linked to each other, the surface area reflects exchange capacity with the environment (i.e. gas exchange with the atmosphere), whereas the volume is linked to the internal redistribution of energy resources. Thin stems and branches of trees imply a larger surface area of metabolically active wood (cambium and phloem cells) for a given volume of sapwood parenchyma[2]. Thus, surface area per unit volume is linked to the tree controls of internal physiological processes. A large proportion of carbon resulting from autotrophic respiration associated with maintenance and growth processes of trees is released to the atmosphere through stems and branches[3–5], with larger surface area per unit volume linked to higher woody CO_2, methane, N_2O and volatile organic compounds effluxes[6–8]. Small diameter wood such as thinner branches can account for most of the total woody tissue CO_2 efflux in Costa Rican tropical forests[2]. The CO_2 efflux of small branches may be linked to the larger proportion of live cells in the inner bark in comparison to the proportion of live cells in the xylem[9]. Thereby, CO_2-budget models of plant growth and carbon dynamics are based on whole-tree respiration rates in relation to plant surface area[10,11].

This relationship has also large effects on the tree interaction with external biophysical elements. In particular, the wood surface area per unit volume is directly linked to the amount of habitat for plants, microorganisms, insects, birds, and mammals in forest canopies[12], and modifies biophysical variables, including wind speed, light intensity[13] and rainfall storage capacity[14].

Path fraction: What are the lengths of paths from tree base to each branch tip? TLS provides indirect measurements of path length and parent-child relations of the branches to calculate the number of terminal branches. The mean path length divided by the maximum path length of a tree provides measures of an architectural metric known as path fraction[15,16]. The path length is the length that water needs to travel to reach the leaves, and thus path fraction is directly related to hydraulic conductivity and nutrient transportation that ultimately may limit the plant photosynthetic capacity[16].

Path fraction was calculated from the QSMs using the *calculate_PathFraction* function in TreeQSM_Architecture (https://github.com/TobyDJackson/TreeQSM_Architecture). Path fraction varies between 0 and 1, with values of 1 indicating that the tree has a small number of twigs and all the path lengths are equal. An increase in path fraction is linked to a reduced hydraulic conductivity, because the average transport distance from trunk to twig increases[16]. Thereby, high values represent umbrella-shaped crowns that prioritize sun exposure and light capture but is structurally expensive to build and hydraulically inefficient[17], as trees with longer water and nutrient transport distances can require more construction tissue[16].

Asymmetry: Trees were split into eight segments centered around the stem, and stems and branches woody volume were summed. The maximum volume observed among the 8 segments by the mean volume represents a measure of crown asymmetry[18]. Asymmetry was calculated from the QSMs using the function *calculate_CrownAsymmetry* in TreeQSM_Architecture (https://github.com/TobyDJackson/TreeQSM_Architecture).

There is evidence that crown asymmetry is an adaptation to maximise solar radiation capture by shifting their trunks and branches towards canopy gaps or away from their neighbours to avoid competition[19–21]. Thus, competition pressure may induce crown asymmetry of supressed trees

competing for light[22]. However, crown asymmetry is linked to regulation of the mechanical stability of trees, with asymmetrical crowns more vulnerable to wind[23,24]. The mechanical stability of trees is of particular importance for tall trees in fragmented forests, where canopy openness leads to higher air turbulence caused by strong winds and exposure of tall trees to wind[25,26]. Indeed, asymmetrical trees in forest edges can be vulnerable to wind and leads to higher risks of snapping and uprooting during wind[27], which is a strong driver of tree mortality in Amazonian forests[28].

Relative crown dimensions: Here, crown is defined as the upper part of a tree from the first major branch and also based on the horizontal distance from the base to the tip divided by DBH, namely crown reach. A major branch is defined as a branch whose diameter is larger than 5% of the tree's DBH or larger than 5 cm. The reach is defined as a reach larger than the median reach or 10. Three metrics were derived from the TLS-based crown dimensions: 1) relative crown width, calculated as the average crown diameter by DBH; 2) relative crown depth, calculated as the crown height by tree height; and 3) crown volume.

Investment in crown diameter and crown height represents a trade-off between size and shade-tolerance[29]. Plants in light-limited environments in the forest understory tend to have shallow broad crowns with minimal leaf overlap to maximise light capture and withstand falling debris, which leads to positive increments in growth[30–32]. Trees growing in the light-richer environments of forest edges may be able to invest in vertical crown growth to better compete with neighbours that grow taller[29], despite the higher self-shading within crown[33]. However, the lower soil moisture and higher temperatures of forest edges may affect crown growth, with crowns becoming smaller in diameter to reduce path lengths[34]. However, regardless of the environmental conditions driving crown variability, crown dimensions depend on the proportion of biomass allocated to the branches[35], with effects on the growth and mortality of trees[36,37].

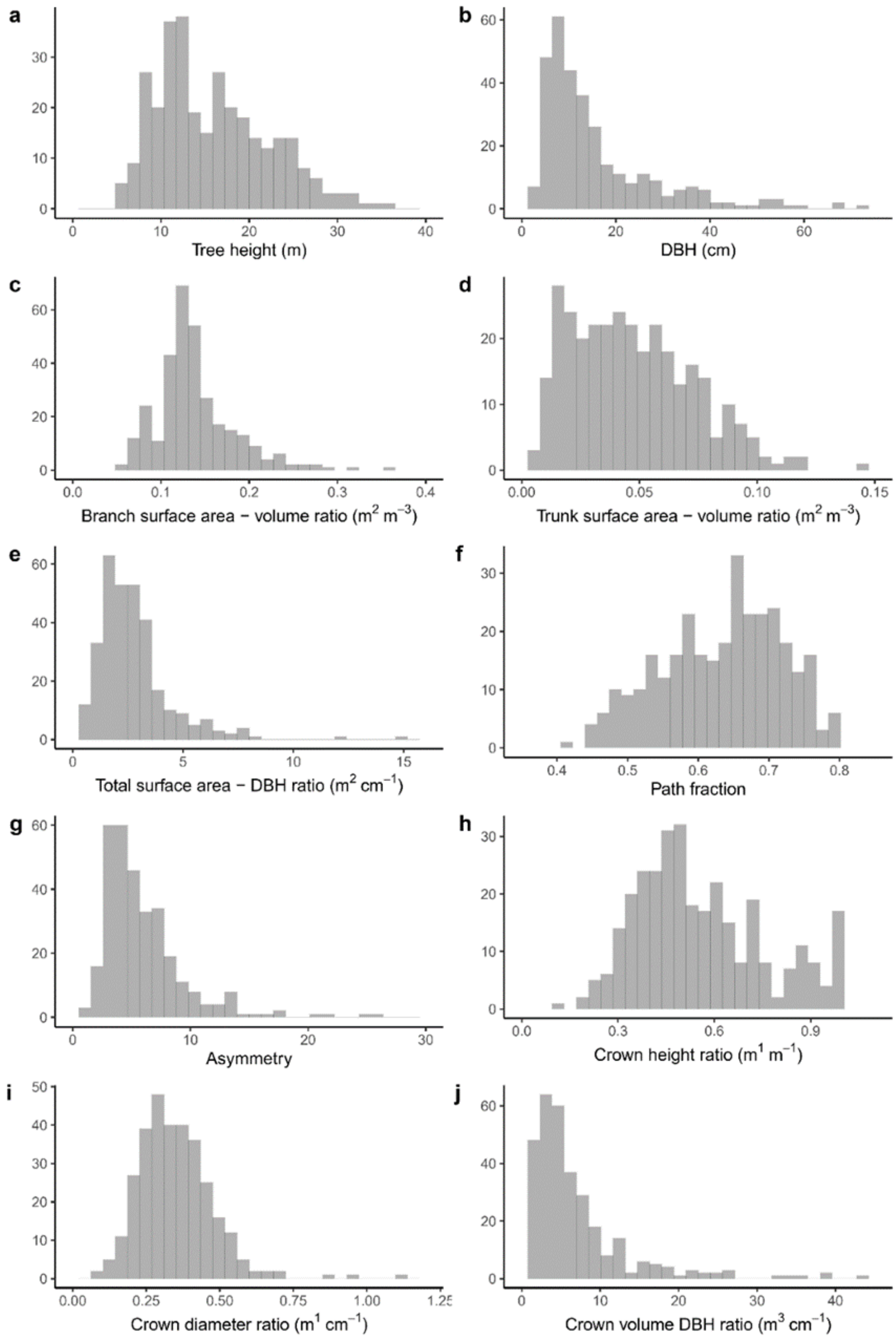

Supplementary Figure 2. Distribution of architectural traits estimated from Quantitative Structural Modelling (QSM) for 315 trees in Central Amazonia.

Supplementary methods 3: Tree size and year of recruitment

Changes in tree architecture may arise from a combination of phenotypic plasticity as a response to changes in microclimatic conditions and light abundance, as well as shifts in species composition that prioritise species that are more adapted to these conditions. Considering that the forest fragments of our study area were isolated in 1979, with the first tree measurements in 1980, we may expect that the majority of the short trees of our study measured in 2019 using TLS comprise of recruits that have colonised the area after the start of the project, whereas upper canopy and emergent trees may have been in the area much longer than the establishment of the fragmentation experiment. Using tree height above 20 m as a reference point to designate taller trees, long-term field measurements of a subset (N = 112) of our TLS dataset show that tall trees have been first measured during the initial years of the BFFDP experiment before 1990, which indicates that tall trees are those that have survived the fragmentation effects (Supplementary Figure 3). Thereby changes in the architecture of tall trees may indicate how the structure of plants may change as a result of acclimation or adaptation. However, our results also indicate that ~ 33% of trees smaller than 20 m in height have also been in the area during the initial stages of the BDFFP experiment, which suggests that not all small trees are new, young recruits, but also include slow-growing trees that may have created mechanisms of adaptation to edge effects.

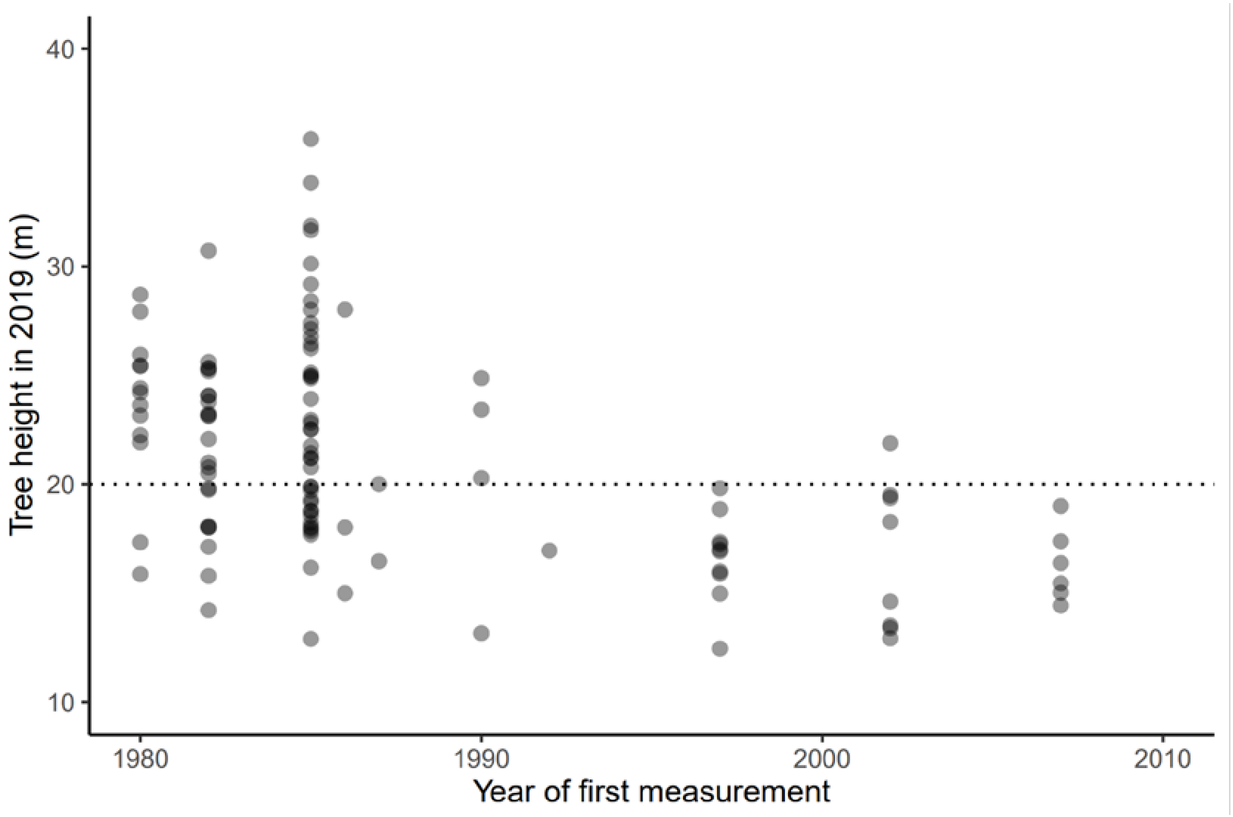

Supplementary Figure 3. Tree height of 112 trees and year of their first DBH measurement in the field (once these trees reached ≥ 10 cm in DBH). These results provide a perspective of tree height and their ontogenetic stage to which trees were subject to fragmentation effects.

Supplementary methods 4: Extent of edge effects on architectural metrics

To estimate the extent of edge effects on each architectural trait, we used mixed linear models (LME, Eq. 1) that contained a variable representing the plot category of location near an edge or in the forest fragment interior (edge effects), following Nunes and colleagues[38]. The model also included a variable that represented tree height (H), considering that architectural traits co-vary with tree size and ontogenetic stage[17,39]. Edge effects and tree height were treated as additive terms to examine the significance of fragmentation and tree height on the variation of architectural traits. We also included an interaction term edge effects × tree height, as fragmentation may have different effects on trees of different heights (Eq. 5). To examine the influences of distance from edges on the allometric relationships between woody volume and DBH^2 H or woody volume and DBH, we tested how the log transformed variables interacted with edge effects (Eq. 6 and Eq. 7).

Nested effects of forest site (Colosso versus Dimona sites), fragment size (1, 10 or 100 ha) and plot (or transect) identity were treated as random variables (μ), allowing us to account for the nested spatial variation in architectural traits and to include any idiosyncratic differences between forest site, fragment size and micro-environmental variation (i.e., topography, soil) between plots.

We then tested the influence of distance to edges on each architectural trait with distances to edges varying between 1 and 100 m, including tree woody volume. We then tested the influence of distance to edges, varying from 1 to 100 m, on each architectural trait; we determined the edge effects extent for each architectural trait based on the maximum absolute t-value of the term "edge effects" of the model (Supplementary Figure 4). The edge effects extent was then used to categorise our analysis into edge versus interior trees during all trait analyses.

The distance by which trees have been affected varies depending on the architectural attribute and hence the number of trees classified as edge versus intact was dependent on the architectural metric (Supplementary Figure 4). Surface area-to-volume ratio of the branches was sensitive to the first 38 m from forest edges (t = 5.568; P-value = 0), varied with tree height (t = -8.91; P-value = 0) and had a statistically significant interaction between tree height and edge effects (t = -5.27; P-value = 0). Surface area-to-volume ratio of the trunk was sensitive to the first 47 m from forest edges (t = -1.77; P-value = 1e-03), varied with tree height (t = -26.15; P-value = 0) and had a statistically significant interaction between tree height and edge effects (t = 3.58; P-value = 4e-04). Path fraction was sensitive to the first 19 m from forest edges (t = -2.22; P-value = 0.0267), did not vary with tree height (t = 1.5636; P-value = 0.1189) and had a significant interaction between tree height and edge effects (t = 2.20109; P-value = 0.0285). Asymmetry was sensitive to the first 55 m from forest edges (t = -3.22; P-value = 0.0014), varied with tree height (t = 17.98; P-value = 0. 0000) and had a significant interaction between tree height and edge effects (t = 3.73; P-value = 0.0002). Relative crown width was also sensitive to the first 10 m from forest edges (t = 2.538; P-value = 0.0116), varied with tree height (t = -3.1849; P-value = 0.0016), but did not have a statistically significant interaction between tree height and edge effects (t = -1.688; P-value = 0.0924). Relative crown height was sensitive to the first 10 m from forest edges (t = -2.22; P-value = 0.0270), did not vary with tree height (t = -1.63; P-value = 0.1027) and had no interaction between tree height and edge effects (t = 1.734; P-value = 0.0838).

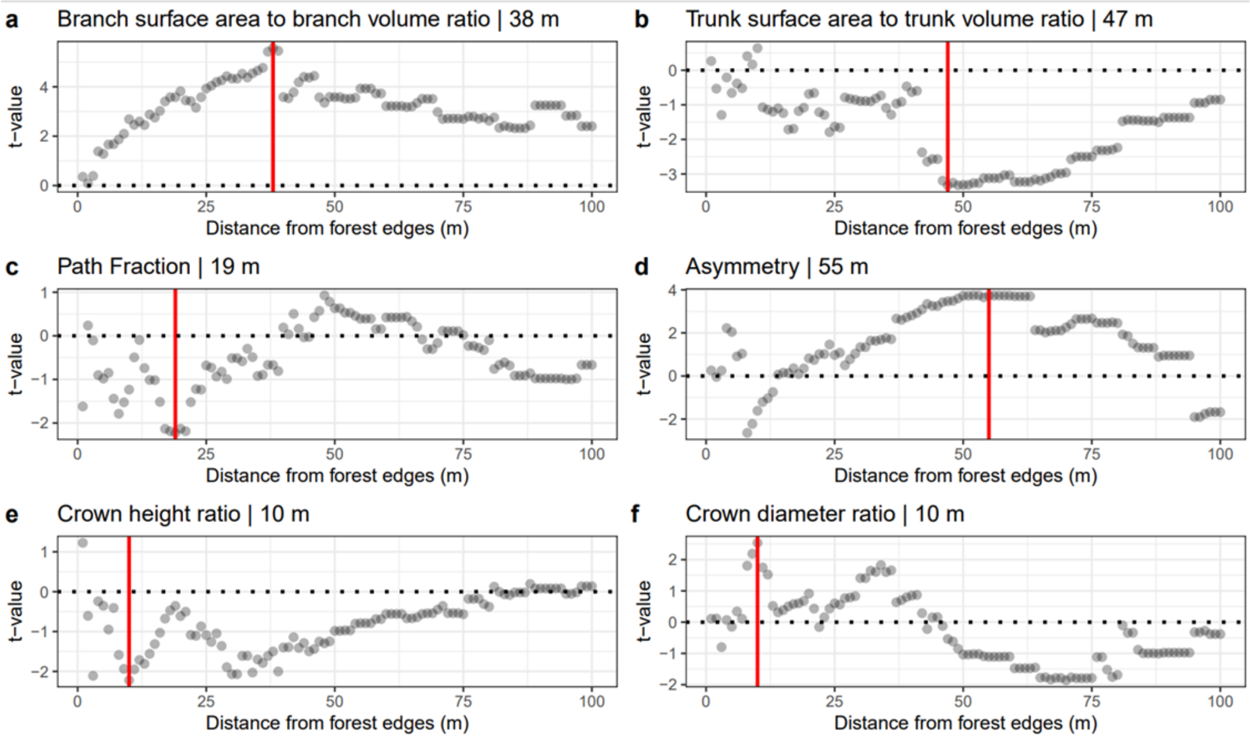

Supplementary Figure 4. Edge effects extent can vary for each architectural metric. We demonstrate the edge effects size as the maximum or minimum local t-value in metres (vertical red line) from mixed linear models.

Supplementary methods 5: Partitioning of variance

The spatial position with plot identity nested within landscape, and landscape nested within forest region had effects that varied depending on the architectural metric (Supplementary Figure 5). We examined the explained variance by the random variables of Eq.5 to investigate the spatial variability of architectural traits arising from region, landscape and plot. The LME models were fitted using the lme function in the “nlme” R package.

Region - Colosso versus Dimona - had relatively large effects on the relative crown height and path fraction. Landscape, seen as the position of the fragment in the landscape alongside the effects of fragment size, had particularly large effects on the surface area per unit volume of branches. Plot identity, with small-scale variations such as topography and soil, influenced most variables, with a particular influence on tree asymmetry. The within-plot variability, that can be seen as local effects arising from edge effects, as well as species-specific and ontogenetic influences accounted

for most of the trait variability (50 – 85%). The within-plot variability also includes analytical errors, such as those arising from measurement, co-registration, tree extraction and QSM – although we were unable to quantify them. The results may help elucidate the generality of our findings in architectural variation across Central Amazonia, although large-scale Amazonian gradients in topography, edaphic properties and climate remain to be tested.

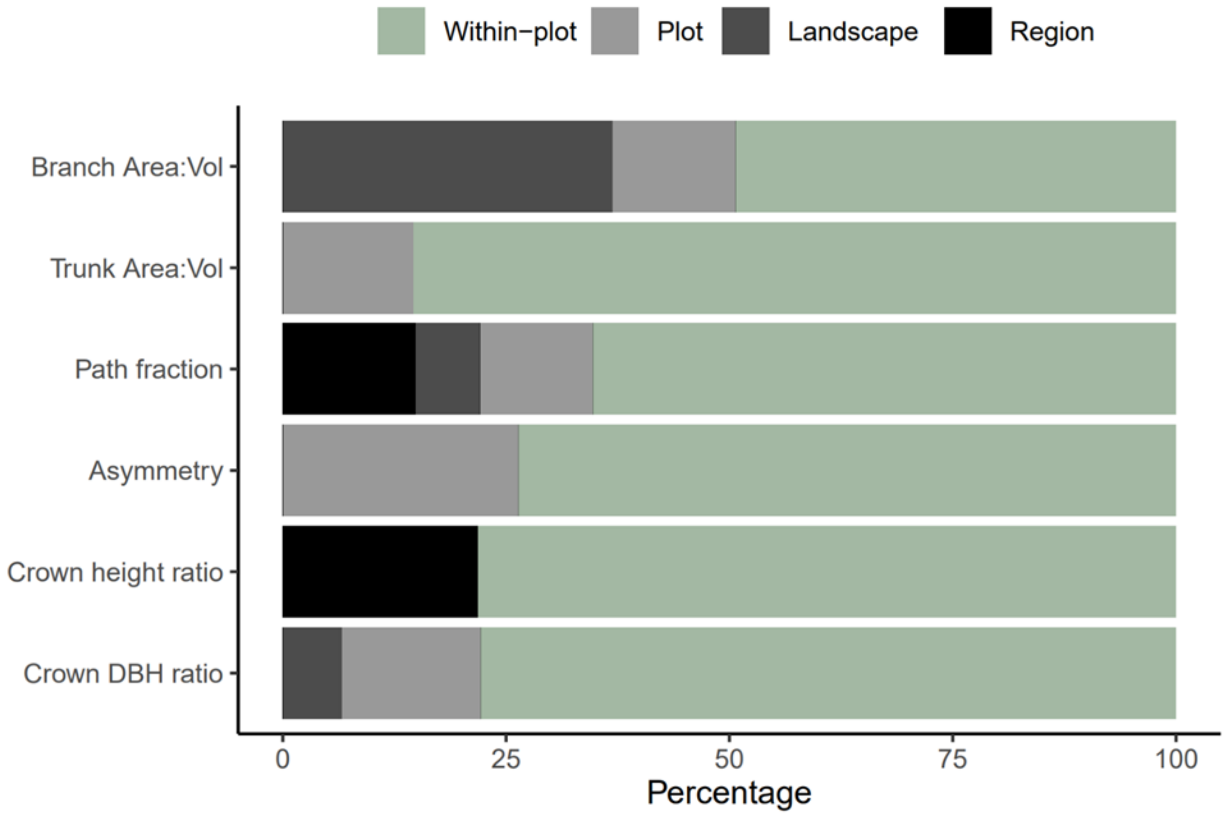

Supplementary Figure 5. Partitioning of variance using mixed-effects models. We used the variance explained by the random variables of Eq.1 to investigate the spatial variability of architectural metrics arising from region, landscape and plot. The results may help elucidate the generality of our findings in architectural variation across Central Amazonia, although large-scale Amazonian gradients in topography, edaphic properties and climate remain to be tested. The spatial position with plot identity nested within landscape, and landscape nested within forest region had effects that varied depending on the architectural metric.

Supplementary methods 6: Correlation among architectural traits

A correlation matrix (Supplementary Figure 6) shows that for a given woody volume, trees that have high surface area in the trunks tend to have high surface area in the branches (r = 0.76). These high surface area trees tend to be more symmetrical (-0.94, -0.81) and have lower path fraction (-0.73, -0.64). Our results show that wider crowns relative to their stem size are more symmetrical and have higher path fraction. These results indicate that trees that afford to have lengthier branches to have a large horizontal crown growth tend to favour traits that provide mechanical stability and hydraulic efficiency.

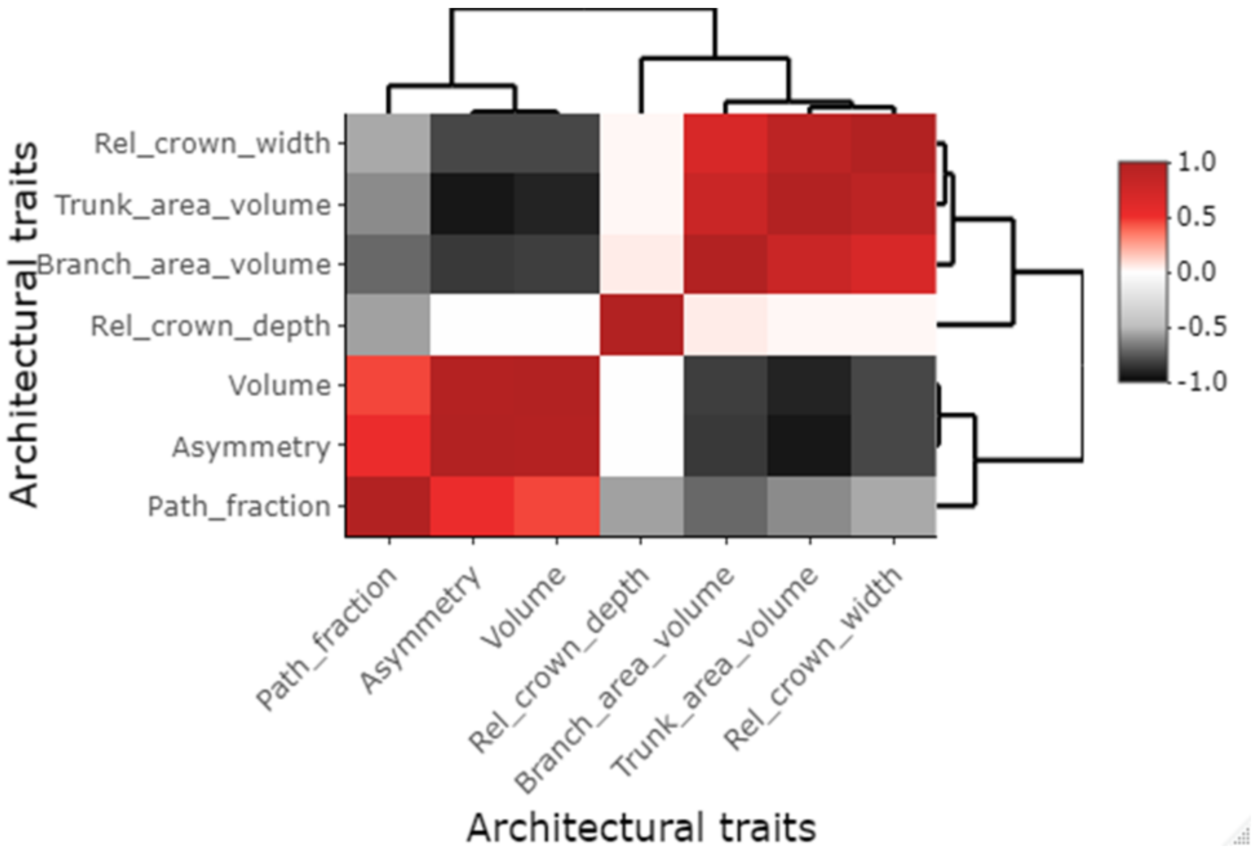

Supplementary Figure 6. Correlation analysis of six architectural traits and woody volume using hierarchical clustering. Red indicates positive correlation and black indicates negative correlation.

Tree architecture reflects a combination of architectural traits that co-vary in Central Amazonian forests. We demonstrate that a principal component analysis decomposed in two independent axes explained 65% of the total variation (Supplementary Figure 7a). The first component explained 43.7% of the total variation in tree architecture, and was tightly linked to variation in the woody volume. These results indicate that variation our chosen architectural metrics had a strong link with the woody volume of individual trees. Trees with higher woody volume have thicker trunks and branches, and have traits that maximise light capture, such as higher asymmetry and higher path fraction (Supplementary Figure 7b).

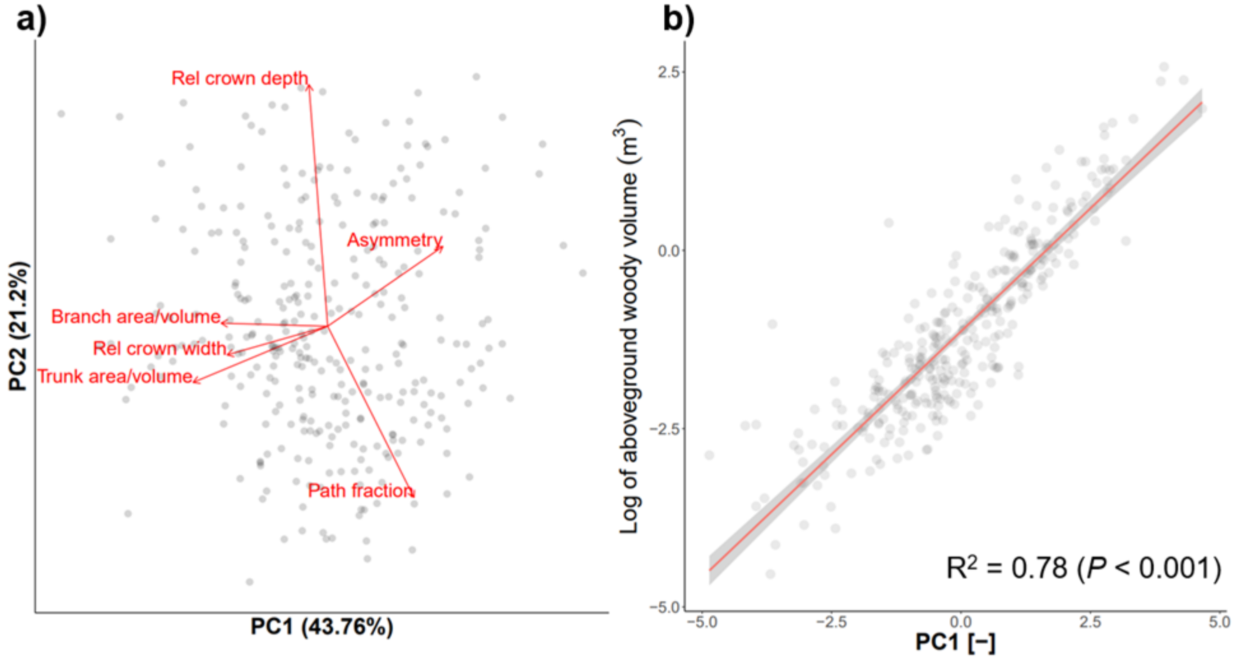

Supplementary Figure 7. Principal component analysis indicating the multidimensional space of tree architecture. Two independent principal components explain 2/3 of the total trait variation. The first component, PC1, explains 43% is tightly linked to the logarithm of aboveground woody volume (R^2 = 0.78, P-value < 0.001).

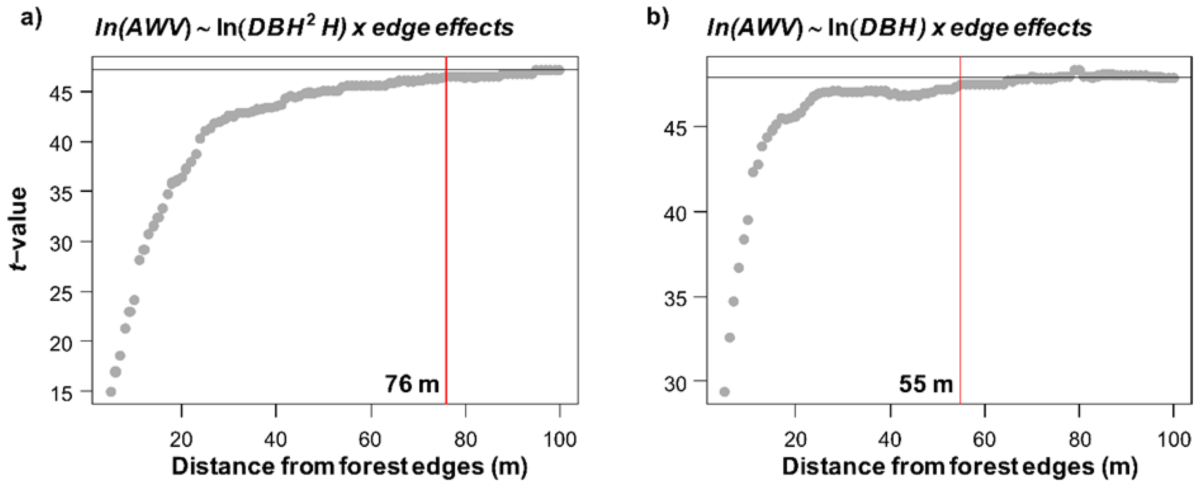

Supplementary Figure 8. Relationship between distance from edges in m and the t-value for the mixed-effects models that predict the logarithm of aboveground woody volume as a function of a) the logarithm of $DBH^2 H$ (cm m^2) and b) the logarithm of DBH (cm). DBH is the diameter at breast height measured at 1.3 m above the ground in cm, and H is the total tree height in metres. Each point represents the t-value of the interaction between the independent variable and edge effects, with the latter as a categorical variable representing the plot category of location near an edge or in the forest interior. Edge effects extent is denoted as a vertical red line, when the t-value saturates and no significant improvement of the model is observed (black horizontal line).

Supplementary Table 1. Allometric model specification and comparison. Results from linear models: ln (W*oody volume*) ~ ln (*DBH*^{2} x *Tree Height*) and ln (*Woody volume*) ~ ln (*DBH*). Let Y = Woody volume (m^3); x = DBH^2 x H or DBH, where DBH is the stem diameter at breast height measured at 1.3 m height in cm and H the total tree height in m. We compared these linear models with linear models that included a quadratic term of the independent variable to include potential effects of time of establishment (surviving trees versus colonising recruits) on the allometric relationships. Model explanatory power was assessed in terms of AIC. The model with the lowest AIC for interior and edges was selected (in bold).

Model	DBH^2 x Tree Height		DBH	
	Interior	Edge	Interior	Edge
Y ~ X	**70.5**	292.4	**71.9**	**308.3**
Y ~ X + X^2	72.4	**290.4**	73.7	309.0

Supplementary Table 2: Estimates of parameters β_0, β_1 and β_2, alongside their *P*-values, obtained using mixed-effects models: ln (W*oody volume*) ~ ln (*DBH*^{2} x *Tree Height*) and ln (*Woody volume*) ~ ln (*DBH*). DBH is the stem diameter at breast height measured at 1.3 m in height. R^2 here is used as a statistics to depict goodness-of-fit of each model.

Parameters	DBH^2 x Tree Height		DBH	
	Interior	Edge	Interior	Edge
β_0 (*P*-value)	-0.2166 (<0.001)	-0.1354 (<0.001)	2.81307 (<0.001)	2.4798 (<0.001)
β_1 (*P*-value)	0.8114 (<0.001)	0.7240 (<0.001)	1.99525 (<0.001)	1.7337 (<0.001)
β_2 (*P*-value)		0.0162 (0.0491)		
R^2	0.8956	0.8882	0.8832	0.8687

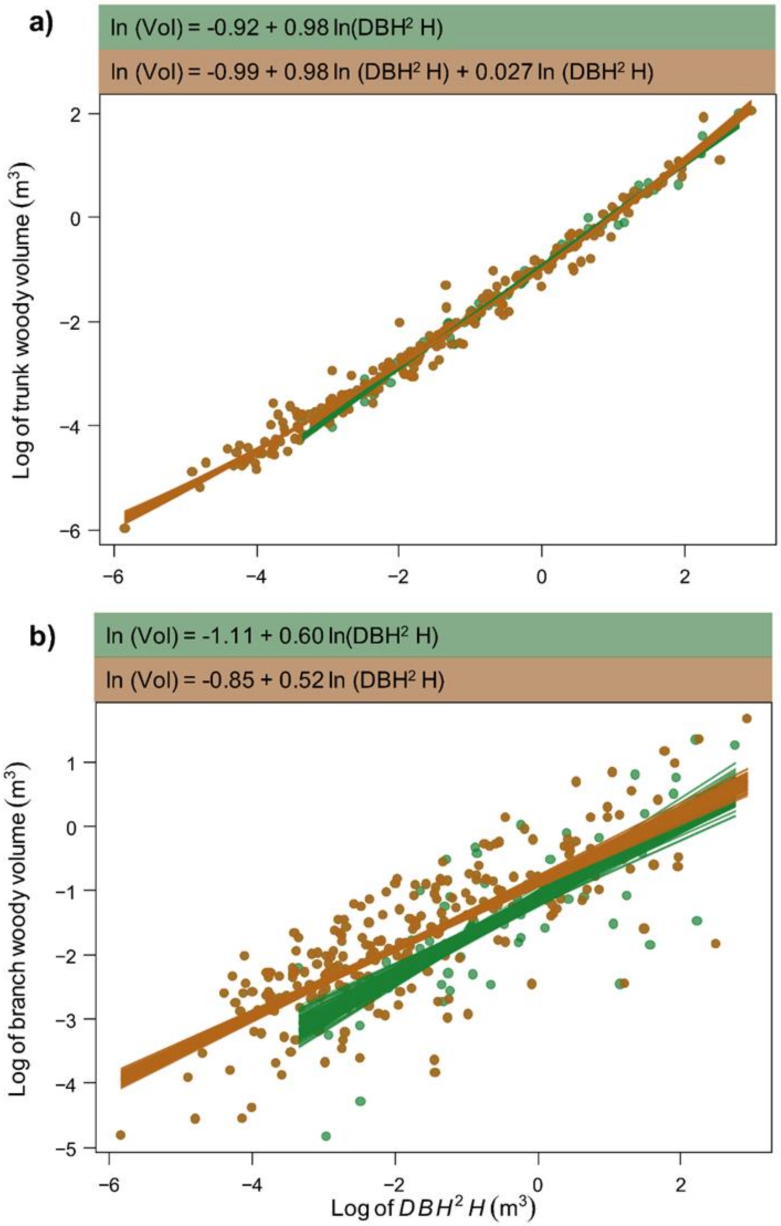

Supplementary Figure 9: Linear mixed model regressions between the logarithm of DBH^2 H (m^3) and a) the logarithm of trunk volume (m^3) and b) the logarithm of branch volume for trees in forest edges (orange) and forest interior (green). Let volume in m^3, diameter at breast height (DBH) in cm and tree height (H) in m. Plot identity nested within landscape (position of fragment within the landscape and fragment size) and region within Central Amazonia were included as random variables. Each point represents an observed value and each line corresponds to the model prediction obtained by fitting 200 randomised permutations of subsets split into 80/20 for calibration and validation, respectively.

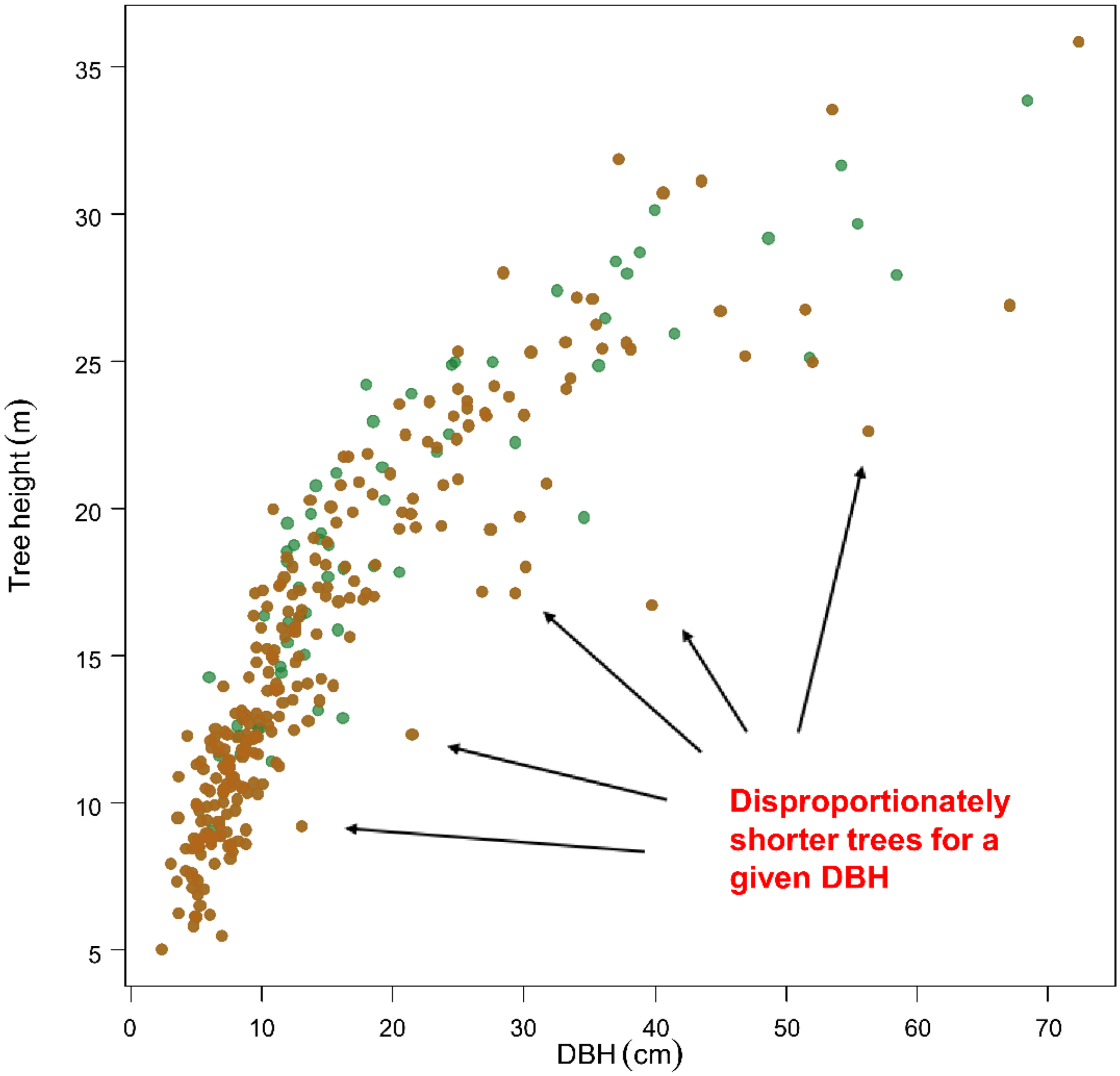

Supplementary Figure 10: Diameter at breast height (DBH, cm) versus tree height (m) for trees in forest edges (orange) and forest interior (green). Tree measurements were acquired using a Terrestrial Laser Scanner (TLS) within the Biological Dynamics of Forest Fragments Project (BDFFP) in Central Amazonia. This relationship demonstrates that some trees are disproportionately shorter for a given DBH in the edges, which may reflect damages that affect tree height common near forest edges or selective forces that have favoured trees with lower height. Here, we did not attempt to predict tree height as a function of DBH to avoid ignoring these disproportionally shorter trees. However, we do acknowledge them as a potential cause for the smaller volume of tall trees near the edge when volume is predicted as a function of DBH only.

SUPPLEMENTARY REFERENCES

1. Arseniou, G., MacFarlane, D. W. & Seidel, D. Woody Surface Area Measurements with Terrestrial Laser Scanning Relate to the Anatomical and Structural Complexity of Urban Trees. *Remote Sensing* **13**, 3153 (2021).

2. Cavaleri, M. A., Oberbauer, S. F. & Ryan, M. G. Wood CO2 efflux in a primary tropical rain forest. *Glob. Chang. Biol.* **12**, 2442–2458 (2006).

3. Chambers, J. Q. *et al.* Respiration from a tropical forest ecosystem: Partitioning of sources and low carbon use efficiency. *Ecol. Appl.* **14**, 72–88 (2004).

4. Trumbore, S. Carbon respired by terrestrial ecosystems - recent progress and challenges. *Glob. Chang. Biol.* **12**, 141–153 (2006).

5. Susan E. Trumbore, Alon Angert, Norbert Kunert, Jan Muhr & Jeffrey Q. Chambers. What's the flux? Unraveling how CO_2 fluxes from trees reflect underlying physiological processes. *New Phytol.* **197**, 353–355 (2013).

6. Robertson, A. L. *et al.* Stem respiration in tropical forests along an elevation gradient in the Amazon and Andes. *Glob. Chang. Biol.* **16**, 3193–3204 (2010).

7. Barba, J. *et al.* Methane emissions from tree stems: a new frontier in the global carbon cycle. *New Phytol.* **222**, 18–28 (2019).

8. Welch, B., Gauci, V. & Sayer, E. J. Tree stem bases are sources of CH4 and N2 O in a tropical forest on upland soil during the dry to wet season transition. *Glob. Chang. Biol.* **25**, 361–372 (2019).

9. Wittmann, C. *et al.* Stem CO2 release under illumination: corticular photosynthesis, photorespiration or inhibition of mitochondrial respiration? *Plant Cell Environ.* **29**, 1149–1158 (2006).

10. Watanabe, T. *et al.* Developing a multilayered integrated numerical model of surface physics â growing plants interaction (MINoSGI). *Glob. Chang. Biol.* **10**, 963–982 (2004).

11. Price, C. A., Enquist, B. J. & Savage, V. M. A general model for allometric covariation in botanical form and function. *Proc. Natl. Acad. Sci. U. S. A.* **104**, 13204–13209 (2007).

12. Nadkarni, N. M. Diversity of Species and Interactions in the Upper Tree Canopy of Forest Ecosystems1. *Integr. Comp. Biol.* **34**, 70–78 (2015).

13. Niinemets, Ü. A review of light interception in plant stands from leaf to canopy in different plant functional types and in species with varying shade tolerance. *Ecol. Res.* **25**, 693–714 (2010).

14. Santos Terra, M. de C. N. *et al.* Stemflow in a neotropical forest remnant: vegetative determinants, spatial distribution and correlation with soil moisture. *Trees* **32**, 323–335 (2018).

15. Bentley, L. P. *et al.* An empirical assessment of tree branching networks and implications for plant allometric scaling models. *Ecol. Lett.* **16**, 1069–1078 (2013).

16. Smith, D. D. *et al.* Deviation from symmetrically self-similar branching in trees predicts altered hydraulics, mechanics, light interception and metabolic scaling. *New Phytol.* **201**, 217–229 (2014).

17. Malhi, Y. *et al.* New perspectives on the ecology of tree structure and tree communities through terrestrial laser scanning. *Interface Focus* **8**, 20170052 (2018).

18. Jackson, T. *et al.* An architectural understanding of natural sway frequencies in trees. *J. R. Soc. Interface* **16**, 20190116 (2019).

19. Berezovskaya, F. S., Karev, G. P., Kisliuk, O. S., Khlebopros, R. G. & Tsel'niker, Y. L. A fractal approach to computer-analytical modelling of tree crowns. *Trees* **11**, 323–327 (1997).

20. Getzin, S. & Wiegand, K. Asymmetric tree growth at the stand level: Random crown patterns and the response to slope. *For. Ecol. Manage.* **242**, 165–174 (2007).

21. Olivier, M.-D., Robert, S. & Fournier, R. A. Response of sugar maple (Acer saccharum, Marsh.) tree crown structure to competition in pure versus mixed stands. *For. Ecol. Manage.* **374**, 20–32 (2016).

22. Young, T. P. & Hubbell, S. P. Crown asymmetry, treefalls, and repeat disturbance of broad-leaved forest gaps. *Ecology* **72**, 1464–1471 (1991).

23. MacFarlane, D. W. & Kane, B. Neighbour effects on tree architecture: functional trade-offs balancing crown competitiveness with wind resistance. *Funct. Ecol.* **31**, 1624–1636 (2017).

24. Jackson, T. *et al.* A new architectural perspective on wind damage in a natural forest. *Front. For. Glob. Chang.* **1**, (2019).

25. Laurance, W. F. & Curran, T. J. Impacts of wind disturbance on fragmented tropical forests: A review and synthesis. *Austral Ecol.* **33**, 399–408 (2008).

26. Vovides, A. G. *et al.* Change in drivers of mangrove crown displacement along a salinity stress gradient. *Funct. Ecol.* **32**, 2753–2765 (2018).

27. Lu, D. *et al.* Detecting dynamics and variations of crown asymmetry induced by natural gaps in a temperate secondary forest using terrestrial laser scanning. *For. Ecol. Manage.* **473**, 118289 (2020).

28. Reis, S. M. *et al.* Climate and crown damage drive tree mortality in southern Amazonian edge forests. *J. Ecol.* **110**, 876–888 (2022).

29. Sheil, D., Salim, A., Chave, J., Vanclay, J. & Hawthorne, W. D. Illumination-Size Relationships of 109 Coexisting Tropical Forest Tree Species. *J. Ecol.* **94**, 494–507 (2006).

30. Valladares, F., Skillman, J. B. & Pearcy, R. W. Convergence in light capture efficiencies among tropical forest understory plants with contrasting crown architectures: a case of morphological compensation. *Am. J. Bot.* **89**, 1275–1284 (2002).

31. Joseph Wright, S. *et al.* Reproductive size thresholds in tropical trees: variation among individuals, species and forests. *J. Trop. Ecol.* **21**, 307–315 (2005).

32. Iida, Y. *et al.* Linking size-dependent growth and mortality with architectural traits across 145 co-occurring tropical tree species. *Ecology* **95**, 353–363 (2014).

33. Pearcy, R. W., Muraoka, H. & Valladares, F. Crown architecture in sun and shade environments: assessing function and trade-offs with a three-dimensional simulation model. *New Phytol.* **166**, 791–800 (2005).

34. Jacobs, M., Rais, A. & Pretzsch, H. How drought stress becomes visible upon detecting tree shape using terrestrial laser scanning (TLS). *For. Ecol. Manage.* **489**, 118975 (2021).

35. Kunz, M. *et al.* Neighbour species richness and local structural variability modulate aboveground allocation patterns and crown morphology of individual trees. *Ecol. Lett.* **22**, 2130–2140 (2019).

36. Sapijanskas, J., Paquette, A., Potvin, C., Kunert, N. & Loreau, M. Tropical tree diversity enhances light capture through crown plasticity and spatial and temporal niche differences. *Ecology* **95**, 2479–2492 (2014).

37. Arellano, G., Medina, N. G., Tan, S., Mohamad, M. & Davies, S. J. Crown damage and the mortality of tropical trees. *New Phytol.* **221**, 169–179 (2019).

38. Nunes, M. H. *et al.* Recovery of logged forest fragments in a human-modified tropical landscape during the 2015-16 El Niño. *Nat. Commun.* **12**, 1526 (2021).

39. Verbeeck, H. *et al.* Time for a plant structural economics spectrum. *Front. For. Glob. Chang.* **2**, (2019).
