# Supplementary material for: Edge effects on tree architecture exacerbate biomass loss of fragmented Amazonian forests
Source: Nat Commun. 2023 Dec 14;14:8129. doi: 10.1038/s41467-023-44004-5 (PMC10721830; doi:10.1038/s41467-023-44004-5)
Supplement: Supplementary file 3 — Reporting Summary [file 41467_2023_44004_MOESM3_ESM.pdf]

nature portfolio

Corresponding author(s): Matheus Henrique Nunes

Last updated by author(s): Nov 16, 2023

Reporting Summary

Nature Portfolio wishes to improve the reproducibility of the work that we publish. This form provides structure for consistency and transparency in reporting. For further information on Nature Portfolio policies, see our Editorial Policies and the Editorial Policy Checklist.

Statistics

For all statistical analyses, confirm that the following items are present in the figure legend, table legend, main text, or Methods section.

n/a	Confirmed	
☐	☒	The exact sample size (*n*) for each experimental group/condition, given as a discrete number and unit of measurement
☒	☐	A statement on whether measurements were taken from distinct samples or whether the same sample was measured repeatedly
☐	☒	The statistical test(s) used AND whether they are one- or two-sided<br>*Only common tests should be described solely by name; describe more complex techniques in the Methods section.*
☐	☒	A description of all covariates tested
☒	☐	A description of any assumptions or corrections, such as tests of normality and adjustment for multiple comparisons
☐	☒	A full description of the statistical parameters including central tendency (e.g. means) or other basic estimates (e.g. regression coefficient) AND variation (e.g. standard deviation) or associated estimates of uncertainty (e.g. confidence intervals)
☐	☒	For null hypothesis testing, the test statistic (e.g. *F*, *t*, *r*) with confidence intervals, effect sizes, degrees of freedom and *P* value noted<br>*Give P values as exact values whenever suitable.*
☒	☐	For Bayesian analysis, information on the choice of priors and Markov chain Monte Carlo settings
☐	☒	For hierarchical and complex designs, identification of the appropriate level for tests and full reporting of outcomes
☒	☐	Estimates of effect sizes (e.g. Cohen's *d*, Pearson's *r*), indicating how they were calculated

Our web collection on statistics for biologists contains articles on many of the points above.

Software and code

Policy information about availability of computer code

Data collection	Measurements of tree architecture were collected using a terrestrial laser scanner (TLS) RIEGL VZ-400i
Data analysis	RiSCAN PRO software version 2.9 provided by RIEGL was used to co-register all the data points collected using the TLS;<br><br>Matlab version R2020a for automatic segmentation using the shortest paths method by Raumonen and colleagues (Raumonen, P., Brede, B., Lau, A. & Bartholomeus, H. A Shortest Path Based Tree Isolation Method for UAV LiDAR Data. in 2021 IEEE International Geoscience and Remote Sensing Symposium IGARSS 724–727 (2021). doi:10.1109/IGARSS47720.2021.9554346);<br><br>RiSCAN PRO software version 2.9 provided by RIEGL was used correct for the automatic segmentation of individual trees;<br><br>Matlab version R2020a to estimate geometric primitives of trees using quantitative structure model (QSM) developed by Raumonen and colleagues (Raumonen, P. et al. Fast Automatic Precision Tree Models from Terrestrial Laser Scanner Data. Remote Sensing 5, 491–520 (2013);<br><br>The following packages in the Software R Core Team (2018) were used for all the statistical analysis: R.matlab ; lidR; nlme ; BIOMASS.

For manuscripts utilizing custom algorithms or software that are central to the research but not yet described in published literature, software must be made available to editors and reviewers. We strongly encourage code deposition in a community repository (e.g. GitHub). See the Nature Portfolio guidelines for submitting code & software for further information.

Data

Policy information about availability of data

All manuscripts must include a data availability statement. This statement should provide the following information, where applicable:

- Accession codes, unique identifiers, or web links for publicly available datasets
- A description of any restrictions on data availability
- For clinical datasets or third party data, please ensure that the statement adheres to our policy

The architectural traits data generated in this study have been deposited in the national Finnish Fairdata services database under accession code https://etsin.fairdata.fi/dataset/c71785e6-58ab-4316-a0fc-28236c76bbe7. The point clouds of individual trees can be available by contacting the corresponding authors upon reasonable request within 10days. Measurements of all trees and their corresponding species identification at the plot level are products of the long-term Biological Dynamics of Forest Fragments Project. The interested party should contact William Laurance (bill.laurance@jcu.edu.au), Ana Andrade (asegalin@gmail.com) and Alberto Vicentini (vicentini.beto@gmail.com) for potential access to these data.

Research involving human participants, their data, or biological material

Policy information about studies with human participants or human data. See also policy information about sex, gender (identity/presentation), and sexual orientation and race, ethnicity and racism.

Reporting on sex and gender	NA
Reporting on race, ethnicity, or other socially relevant groupings	NA
Population characteristics	NA
Recruitment	NA
Ethics oversight	NA

Note that full information on the approval of the study protocol must also be provided in the manuscript.

Field-specific reporting

Please select the one below that is the best fit for your research. If you are not sure, read the appropriate sections before making your selection.

☐ Life sciences ☐ Behavioural & social sciences ☒ Ecological, evolutionary & environmental sciences

For a reference copy of the document with all sections, see nature.com/documents/nr-reporting-summary-flat.pdf

Life sciences study design

All studies must disclose on these points even when the disclosure is negative.

Sample size	*Describe how sample size was determined, detailing any statistical methods used to predetermine sample size OR if no sample-size calculation was performed, describe how sample sizes were chosen and provide a rationale for why these sample sizes are sufficient.*
Data exclusions	*Describe any data exclusions. If no data were excluded from the analyses, state so OR if data were excluded, describe the exclusions and the rationale behind them, indicating whether exclusion criteria were pre-established.*
Replication	*Describe the measures taken to verify the reproducibility of the experimental findings. If all attempts at replication were successful, confirm this OR if there are any findings that were not replicated or cannot be reproduced, note this and describe why.*
Randomization	*Describe how samples/organisms/participants were allocated into experimental groups. If allocation was not random, describe how covariates were controlled OR if this is not relevant to your study, explain why.*
Blinding	*Describe whether the investigators were blinded to group allocation during data collection and/or analysis. If blinding was not possible, describe why OR explain why blinding was not relevant to your study.*

Behavioural & social sciences study design

All studies must disclose on these points even when the disclosure is negative.

Study description	*Briefly describe the study type including whether data are quantitative, qualitative, or mixed-methods (e.g. qualitative cross-sectional,*

Study description	*quantitative experimental, mixed-methods case study).*
Research sample	*State the research sample (e.g. Harvard university undergraduates, villagers in rural India) and provide relevant demographic information (e.g. age, sex) and indicate whether the sample is representative. Provide a rationale for the study sample chosen. For studies involving existing datasets, please describe the dataset and source.*
Sampling strategy	*Describe the sampling procedure (e.g. random, snowball, stratified, convenience). Describe the statistical methods that were used to predetermine sample size OR if no sample-size calculation was performed, describe how sample sizes were chosen and provide a rationale for why these sample sizes are sufficient. For qualitative data, please indicate whether data saturation was considered, and what criteria were used to decide that no further sampling was needed.*
Data collection	*Provide details about the data collection procedure, including the instruments or devices used to record the data (e.g. pen and paper, computer, eye tracker, video or audio equipment) whether anyone was present besides the participant(s) and the researcher, and whether the researcher was blind to experimental condition and/or the study hypothesis during data collection.*
Timing	*Indicate the start and stop dates of data collection. If there is a gap between collection periods, state the dates for each sample cohort.*
Data exclusions	*If no data were excluded from the analyses, state so OR if data were excluded, provide the exact number of exclusions and the rationale behind them, indicating whether exclusion criteria were pre-established.*
Non-participation	*State how many participants dropped out/declined participation and the reason(s) given OR provide response rate OR state that no participants dropped out/declined participation.*
Randomization	*If participants were not allocated into experimental groups, state so OR describe how participants were allocated to groups, and if allocation was not random, describe how covariates were controlled.*

Ecological, evolutionary & environmental sciences study design

All studies must disclose on these points even when the disclosure is negative.

Study description	We investigated tree architecture of forests in Central Amazonia with terrestrial laser scanning (TLS, also terrestrial LiDAR) surveys collected in April 2019. We then combined the TLS measurements with tree measurements of stem diameter across 36 1-ha permanent plots to predict the effects of tree architecture on the aboveground biomass of fragmented forests.
Research sample	The study was conducted in Central Amazonian forests (2°20′ S, 60° 05′ W and 2°24′ S, 59° 52′ W) within the Biological Dynamics of Forest Fragments Project (BDFFP), the world's longest-running experimental study of habitat fragmentation. The region has seen notable carbon and biodiversity losses due to forest fragmentation effects and is predicted to be markedly impacted by climatic changes. The pioneering BDFFP project sites are composed of forest fragments originally isolated in 1980 by converting mature forest into cattle pastures. Currently, the matrix is dominated by secondary growth forests, but a 100 m strip surrounding the forest fragments is regularly cleaned by cutting vegetation regrowth to keep the forest fragments isolated. As an experimental control that minimises anthropogenic influences as confounding factors, such as illegal logging, hunting, fire penetration and pollution, the project offers unique insights into ecological and environmental changes in fragmented forests. We selected four fragments in the sites Dimona and Colosso within the BDFFP to investigate tree architecture with varying distances from the fragment edges (0 – 500 m). At the community level, the forest edges of our study are dominated by a high density of early-successional, fast-growth species, because of the elevated tree mortality near forest edges and seed dispersion from degraded neighbouring habitats, while the interior of these fragments comprises of undisturbed primary forests.
Sampling strategy	The scans covered three transects of 100×10m and three transects of 50 × 10 m near the fragment edges and perpendicular to the forest fragment margins. We also included one transect of 30×10m length in the centre of the 100-ha forest fragment, which lies 500m from any fragment margin to ensure sampling of forest interior in the absence of edge effects on tree architecture.<br>To ensure a full 3D representation of the upper canopy, each transect consisted of three scan lines parallel to each other with scans spaced at 5 m intervals within and between lines. The distance between scanning positions was smaller than the 10-40 m usually applied in previous studies to minimize data uncertainties due to occlusion in dense tropical forests and maximize data acquisition in the upper canopy. Given that the RIEGL VZ-400i has a zenith angle range of 30–130°, an additional scan was acquired at each sampling location with the scanner tilted at 90° from the vertical position. This sampling strategy resulted in 1,188 scans across all transects, with a complete sampling of the full hemisphere in each scan location. A total of 315 individual trees were segmented and extracted from the original TLS point clouds.<br><br>Tree measurements of stem size at the plot level to investigate how tree architecture affects the forest aboveground biomass were made within 44 1-ha long-term permanent plots of four reserves (Dimona, Colosso, Florestal and Porto Alegre) in the Biological Dynamics of Forest Fragments Project (BDFFP), the world's longest-running experimental study of habitat fragmentation. The study area is surrounded by large expanses (>200 km) of continuous forest to the west, north, and east. In the early 1980s, fragments were isolated by distances of 80–650 m from surrounding forest by clearing the intervening vegetation to establish cattle pastures. Fragments were fenced to prevent encroachment by cattle. Reserves ranging from 1 to 1000 ha in area were delineated in nearby continuous forest to serve as experimental controls. Because of low pasture productivity, the ranches were gradually abandoned, and 3- to 15-m–tall secondary forests (dominated by Cecropia spp. or Vismia spp.) proliferated in many formerly cleared areas. To help maintain fragment isolation, 100-m–wide strips of regrowth were cleared and burned around each fragment on two or three occasions.
Data collection	TLS-based data were acquired using a RIEGL VZ-400i system in April 2019. Eduardo Eiji Maeda and Matheus Henrique Nunes led the field campaigns.

	Field measurements of stem size and species identification were made in 2015 and 2016 and were led by José Luís Camargo, Ana Andrade, William Laurance, Susan Laurance and Alberto Vincentini.
Timing and spatial scale	TLS campaign occurred in April 2019 for three weeks. Tree measurements of stem size within permanent plots, alongside plant identification, were made for two years during 2015 and 2016.
Data exclusions	No data were excluded from the analysis
Reproducibility	Our experiment did not depend on reproducibility - the results from each measurement were taken into account and used as input data for our prediction models.
Randomization	Plots and regions were treated as random factors in the linear mixed modelling (LME) to include any idiosyncratic differences between plots and regions, with a random intercept term capturing variation in architectural metrics between plots. The LME model was fitted using the lme function in the nlme R package.
Blinding	Blinding is not relevant to our study. No persons were used in the study.

Did the study involve field work? ☒ Yes ☐ No

Field work, collection and transport

Field conditions	Tree measurements were made in April 2019 in remote fragments located 4 hours minimum from Manaus (Brazil) by a 4 x 4 pick up truck. Most days were rainy in the evenings, with sunny mornings that allowed successful collections of data using a terrestrial LiDAR. Temperatures were ideal for collection, varying between 25 and 35 degrees Celsius.
Location	The study is located in Central Amazonia within the Biological Dynamics of Forest Fragments Project. TLS measurements were made in Dimona (2°20′ S, 60° 05′ W) and Colosso (2°24′ S, 59° 52′ W) experimental areas. Tree measurements of stem size at the plot level and plant identification were made within the experimental areas Dimona, Colosso, Porto Alegre and Florestal.
Access & import/export	Permit to collected terrestrial LiDAR data was granted by ICMBio (Instituto Chico Mendes da Biodiversidade), which did not involve import or export of biological materials.<br><br>The Biological Dynamics of Forest Fragments Project (BDFFP) is located within a conservation unit and is a collaborative project between Brazilian National Institute for Amazonian Research (INPA) and the Smithsonian Institution. Logistics, including work permit, are arranged and endorsed by INPA.
Disturbance	No disturbance was caused by data collection using a terrestrial LiDAR.<br><br>Disturbances in the forest to establish forest fragments in continuous forested areas were made in 1980. The project was approved by SUFRAMA (Manaus Free Trade Zone Superintendence) to establish long-term forest monitoring of fragmented forested ecosystems.

Reporting for specific materials, systems and methods

We require information from authors about some types of materials, experimental systems and methods used in many studies. Here, indicate whether each material, system or method listed is relevant to your study. If you are not sure if a list item applies to your research, read the appropriate section before selecting a response.

Materials & experimental systems

n/a	Involved in the study	
☒	☐	Antibodies
☒	☐	Eukaryotic cell lines
☒	☐	Palaeontology and archaeology
☒	☐	Animals and other organisms
☒	☐	Clinical data
☒	☐	Dual use research of concern
☐	☒	Plants

Methods

n/a	Involved in the study	
☒	☐	ChIP-seq
☒	☐	Flow cytometry
☒	☐	MRI-based neuroimaging

Antibodies

Antibodies used	*Describe all antibodies used in the study; as applicable, provide supplier name, catalog number, clone name, and lot number.*
Validation	*Describe the validation of each primary antibody for the species and application, noting any validation statements on the manufacturer's website, relevant citations, antibody profiles in online databases, or data provided in the manuscript.*

Eukaryotic cell lines

Policy information about cell lines and Sex and Gender in Research

Cell line source(s)	*State the source of each cell line used and the sex of all primary cell lines and cells derived from human participants or vertebrate models.*
Authentication	*Describe the authentication procedures for each cell line used OR declare that none of the cell lines used were authenticated.*
Mycoplasma contamination	*Confirm that all cell lines tested negative for mycoplasma contamination OR describe the results of the testing for mycoplasma contamination OR declare that the cell lines were not tested for mycoplasma contamination.*
Commonly misidentified lines (See ICLAC register)	*Name any commonly misidentified cell lines used in the study and provide a rationale for their use.*

Palaeontology and Archaeology

Specimen provenance	*Provide provenance information for specimens and describe permits that were obtained for the work (including the name of the issuing authority, the date of issue, and any identifying information). Permits should encompass collection and, where applicable, export.*
Specimen deposition	*Indicate where the specimens have been deposited to permit free access by other researchers.*
Dating methods	*If new dates are provided, describe how they were obtained (e.g. collection, storage, sample pretreatment and measurement), where they were obtained (i.e. lab name), the calibration program and the protocol for quality assurance OR state that no new dates are provided.*

☐ Tick this box to confirm that the raw and calibrated dates are available in the paper or in Supplementary Information.

Ethics oversight	*Identify the organization(s) that approved or provided guidance on the study protocol, OR state that no ethical approval or guidance was required and explain why not.*

Note that full information on the approval of the study protocol must also be provided in the manuscript.

Animals and other research organisms

Policy information about studies involving animals; ARRIVE guidelines recommended for reporting animal research, and Sex and Gender in Research

Laboratory animals	*For laboratory animals, report species, strain and age OR state that the study did not involve laboratory animals.*
Wild animals	*Provide details on animals observed in or captured in the field; report species and age where possible. Describe how animals were caught and transported and what happened to captive animals after the study (if killed, explain why and describe method; if released, say where and when) OR state that the study did not involve wild animals.*
Reporting on sex	*Indicate if findings apply to only one sex; describe whether sex was considered in study design, methods used for assigning sex. Provide data disaggregated for sex where this information has been collected in the source data as appropriate; provide overall numbers in this Reporting Summary. Please state if this information has not been collected. Report sex-based analyses where performed, justify reasons for lack of sex-based analysis.*
Field-collected samples	*For laboratory work with field-collected samples, describe all relevant parameters such as housing, maintenance, temperature, photoperiod and end-of-experiment protocol OR state that the study did not involve samples collected from the field.*
Ethics oversight	*Identify the organization(s) that approved or provided guidance on the study protocol, OR state that no ethical approval or guidance was required and explain why not.*

Note that full information on the approval of the study protocol must also be provided in the manuscript.

Clinical data

Policy information about clinical studies

All manuscripts should comply with the ICMJE guidelines for publication of clinical research and a completed CONSORT checklist must be included with all submissions.

Clinical trial registration	*Provide the trial registration number from ClinicalTrials.gov or an equivalent agency.*
Study protocol	*Note where the full trial protocol can be accessed OR if not available, explain why.*
Data collection	*Describe the settings and locales of data collection, noting the time periods of recruitment and data collection.*

Outcomes	*Describe how you pre-defined primary and secondary outcome measures and how you assessed these measures.*

Dual use research of concern

Policy information about dual use research of concern

Hazards

Could the accidental, deliberate or reckless misuse of agents or technologies generated in the work, or the application of information presented in the manuscript, pose a threat to:

No	Yes	
☒	☐	Public health
☒	☐	National security
☒	☐	Crops and/or livestock
☒	☐	Ecosystems
☒	☐	Any other significant area

Experiments of concern

Does the work involve any of these experiments of concern:

No	Yes	
☒	☐	Demonstrate how to render a vaccine ineffective
☒	☐	Confer resistance to therapeutically useful antibiotics or antiviral agents
☒	☐	Enhance the virulence of a pathogen or render a nonpathogen virulent
☒	☐	Increase transmissibility of a pathogen
☒	☐	Alter the host range of a pathogen
☒	☐	Enable evasion of diagnostic/detection modalities
☒	☐	Enable the weaponization of a biological agent or toxin
☒	☐	Any other potentially harmful combination of experiments and agents

Plants

Seed stocks	*Report on the source of all seed stocks or other plant material used. If applicable, state the seed stock centre and catalogue number. If plant specimens were collected from the field, describe the collection location, date and sampling procedures.*
Novel plant genotypes	*Describe the methods by which all novel plant genotypes were produced. This includes those generated by transgenic approaches, gene editing, chemical/radiation-based mutagenesis and hybridization. For transgenic lines, describe the transformation method, the number of independent lines analyzed and the generation upon which experiments were performed. For gene-edited lines, describe the editor used, the endogenous sequence targeted for editing, the targeting guide RNA sequence (if applicable) and how the editor was applied.*
Authentication	*Describe any authentication procedures for each seed stock used or novel genotype generated. Describe any experiments used to assess the effect of a mutation and, where applicable, how potential secondary effects (e.g. second site T-DNA insertions, mosiacism, off-target gene editing) were examined.*

ChIP-seq

Data deposition

☐ Confirm that both raw and final processed data have been deposited in a public database such as GEO.

☐ Confirm that you have deposited or provided access to graph files (e.g. BED files) for the called peaks.

Data access links<br>*May remain private before publication.*	*For "Initial submission" or "Revised version" documents, provide reviewer access links. For your "Final submission" document, provide a link to the deposited data.*
Files in database submission	*Provide a list of all files available in the database submission.*
Genome browser session<br>(e.g. UCSC)	*Provide a link to an anonymized genome browser session for "Initial submission" and "Revised version" documents only, to enable peer review. Write "no longer applicable" for "Final submission" documents.*

Methodology

Replicates	*Describe the experimental replicates, specifying number, type and replicate agreement.*
Sequencing depth	*Describe the sequencing depth for each experiment, providing the total number of reads, uniquely mapped reads, length of reads and whether they were paired- or single-end.*
Antibodies	*Describe the antibodies used for the ChIP-seq experiments; as applicable, provide supplier name, catalog number, clone name, and lot number.*
Peak calling parameters	*Specify the command line program and parameters used for read mapping and peak calling, including the ChIP, control and index files used.*
Data quality	*Describe the methods used to ensure data quality in full detail, including how many peaks are at FDR 5% and above 5-fold enrichment.*
Software	*Describe the software used to collect and analyze the ChIP-seq data. For custom code that has been deposited into a community repository, provide accession details.*

Flow Cytometry

Plots

Confirm that:

- [ ] The axis labels state the marker and fluorochrome used (e.g. CD4-FITC).
- [ ] The axis scales are clearly visible. Include numbers along axes only for bottom left plot of group (a 'group' is an analysis of identical markers).
- [ ] All plots are contour plots with outliers or pseudocolor plots.
- [ ] A numerical value for number of cells or percentage (with statistics) is provided.

Methodology

Sample preparation	*Describe the sample preparation, detailing the biological source of the cells and any tissue processing steps used.*
Instrument	*Identify the instrument used for data collection, specifying make and model number.*
Software	*Describe the software used to collect and analyze the flow cytometry data. For custom code that has been deposited into a community repository, provide accession details.*
Cell population abundance	*Describe the abundance of the relevant cell populations within post-sort fractions, providing details on the purity of the samples and how it was determined.*
Gating strategy	*Describe the gating strategy used for all relevant experiments, specifying the preliminary FSC/SSC gates of the starting cell population, indicating where boundaries between "positive" and "negative" staining cell populations are defined.*

- [ ] Tick this box to confirm that a figure exemplifying the gating strategy is provided in the Supplementary Information.

Magnetic resonance imaging

Experimental design

Design type	*Indicate task or resting state; event-related or block design.*
Design specifications	*Specify the number of blocks, trials or experimental units per session and/or subject, and specify the length of each trial or block (if trials are blocked) and interval between trials.*
Behavioral performance measures	*State number and/or type of variables recorded (e.g. correct button press, response time) and what statistics were used to establish that the subjects were performing the task as expected (e.g. mean, range, and/or standard deviation across subjects).*

Acquisition

Imaging type(s) — *Specify: functional, structural, diffusion, perfusion.*

Field strength — *Specify in Tesla*

Sequence & imaging parameters — *Specify the pulse sequence type (gradient echo, spin echo, etc.), imaging type (EPI, spiral, etc.), field of view, matrix size, slice thickness, orientation and TE/TR/flip angle.*

Area of acquisition — *State whether a whole brain scan was used OR define the area of acquisition, describing how the region was determined.*

Diffusion MRI ☐ Used ☐ Not used

Preprocessing

Preprocessing software — *Provide detail on software version and revision number and on specific parameters (model/functions, brain extraction, segmentation, smoothing kernel size, etc.).*

Normalization — *If data were normalized/standardized, describe the approach(es): specify linear or non-linear and define image types used for transformation OR indicate that data were not normalized and explain rationale for lack of normalization.*

Normalization template — *Describe the template used for normalization/transformation, specifying subject space or group standardized space (e.g. original Talairach, MNI305, ICBM152) OR indicate that the data were not normalized.*

Noise and artifact removal — *Describe your procedure(s) for artifact and structured noise removal, specifying motion parameters, tissue signals and physiological signals (heart rate, respiration).*

Volume censoring — *Define your software and/or method and criteria for volume censoring, and state the extent of such censoring.*

Statistical modeling & inference

Model type and settings — *Specify type (mass univariate, multivariate, RSA, predictive, etc.) and describe essential details of the model at the first and second levels (e.g. fixed, random or mixed effects; drift or auto-correlation).*

Effect(s) tested — *Define precise effect in terms of the task or stimulus conditions instead of psychological concepts and indicate whether ANOVA or factorial designs were used.*

Specify type of analysis: ☐ Whole brain ☐ ROI-based ☐ Both

Statistic type for inference — *Specify voxel-wise or cluster-wise and report all relevant parameters for cluster-wise methods.*

(See Eklund et al. 2016)

Correction — *Describe the type of correction and how it is obtained for multiple comparisons (e.g. FWE, FDR, permutation or Monte Carlo).*

Models & analysis

n/a	Involved in the study
☐	☐ Functional and/or effective connectivity
☐	☐ Graph analysis
☐	☐ Multivariate modeling or predictive analysis

Functional and/or effective connectivity — *Report the measures of dependence used and the model details (e.g. Pearson correlation, partial correlation, mutual information).*

Graph analysis — *Report the dependent variable and connectivity measure, specifying weighted graph or binarized graph, subject- or group-level, and the global and/or node summaries used (e.g. clustering coefficient, efficiency, etc.).*

Multivariate modeling and predictive analysis — *Specify independent variables, features extraction and dimension reduction, model, training and evaluation metrics.*
